# Intraoperative 3D imaging in intraarticular tibial plateau fractures - Does it help to improve the patients’ outcomes?

**DOI:** 10.1186/s13018-021-02424-3

**Published:** 2021-05-05

**Authors:** F. Souleiman, R. Henkelmann, J. Theopold, J. Fakler, U. Spiegl, P. Hepp

**Affiliations:** Affiliation: Department of Orthopedics, Trauma and Plastic Surgery, University Hospital Leipzig, Liebigstraße 20, 04103 Leipzig, Germany

**Keywords:** Tibial plateau fracture, 3D imaging, 2D imaging, fluoroscopy, ORIF, Outcome, revision rate, infection

## Abstract

**Background:**

In tibial plateau fractures (TPF) the restoration of an anatomical joint surface as well as an exact subchondral screw position for postoperative stability is crucial for the outcome.

The aim of this study was to determine whether the additional use of an intraoperative 3D imaging intensifier (3D) might help to improve the outcome of complex TPF.

**Methods:**

We performed a retrospective case-control study of a level 1 trauma center. Patients with AO/OTA 41 B3 and C-TPF operated on using a 3D imaging intensifier between November 2015 and December 2018 (3D group) were included. The outcomes of this patients were compared to patients operated without 3D imaging between January 2005 to December 2014 (2D group). The comparison of the groups was performed by matched pair analysis. The functional outcome of both groups was measured by KOOS and Lysholm Score after a follow-up period of at least 12 months. Operation time, infections and postoperative revisions were registered.

**Results:**

In total, 18 patients were included in the 3D group (mean age: 51.0± 16.4 years; 12 females) and an equal number of matching partners from the 2D group (mean age: 50.3± 15.2 years; 11 females) were found (p=0.82; p=0.79). We found 9x B3, 2x C1, 1x C2, 6x C3 fractures according to AO/OTA for each group (p=1.00) with comparable ASA score (p=0.27). The mean operation time was 127.9± 45.9 min and 116.1± 45.7 min for the 3D and 2D group (p=0.28). The mean follow-up time was 20.9± 10.7 months for the 3D and 55.5± 34.7 months for the 2D group (p< 0.001). For the 3D group a mean Lysholm overall score of 67.4± 26.8 and KOOS overall score of 72.6± 23.5 could be assessed. In contrast, a mean Lysholm overall score of 62.0± 21.4 and KOOS overall score of 65.8± 21.6 could be measured in the 2D group (p=0.39; p=0.31). Thereby, functional outcome of the 3D group showed a significant higher KOOS Sport/Rec sub score of 54.7± 35.0 in comparison to the 2D group with 26.7± 31.6 (p= 0.01). Postoperative revisions had to be performed in 27.8% of cases in both groups (p=1.00). Due to the 3D imaging an intraoperative revision was performed in 33.3% (6/18).

**Conclusion:**

In our study we could show that re-reduction of the fracture or implant re-positioning were performed in relevant numbers based on the 3D imaging. This was associated with a midterm clinical benefit in regard to better KOOS Sport/Rec scores.

**Trial registration:**

AZ 488 /20-ek

## Background

Tibial plateau fractures constitute about 1% of all fractures occurring at an incidence of 10.3 per 100,000 annually [1–3]. Arthrosis rates of up to 44 % and rates of revision operations of 25.3 to 45.0% have been described [4, 5]. In addition to fracture severity, the reduction quality is crucial for the development of a posttraumatic arthrosis [6, 7]. For dislocated fractures, surgical therapy is the gold standard [8, 9]. For intraoperative analysis of fracture reduction, conventional fluoroscopy is generally applied. Due to the convexity as well the flat lateral tibial plateau and the concave medial tibial plateau with respective different dorsal slopes, overlaps occur in the lateral fluoroscopy [10, 11]. Complex fracture morphologies are difficult to assess and small joint steps cannot be assessed with sufficient certainty. Postoperative CT imaging is therefore recommended to verify the surgical outcome [12]. If there is a material malposition or an insufficient reduction of the articular surface, a revision operation is necessary. Intraoperative 3D imaging can provide additional information during the operation, which could prevent revision surgeries [13, 14]. There are few studies that support its use in the treatment of intraarticular tibial plateau fractures. Thereby, intraoperative revision rates of up to 26.5% in the treatment of proximal tibial fractures are shown [15–18]. To the authors' knowledge, there is no study comparing the functional outcome after treatment of tibial plateau fractures using intraoperative 3D imaging versus conventional fluoroscopic evaluation using two planes (2D). The aim of this study was to determine whether the additional use of an intraoperative 3D imaging intensifier might help to improve the clinical outcome after surgical treatment of complex tibial plateau fractures. Secondary it was hypothesized, that the rate of postoperative revision surgeries can be reduced.

## Methods

We performed a retrospective case control study at a level 1 trauma center. This study was conducted following approval from the local ethics committee and was performed in accordance with the principles of the Declaration of Helsinki. The consent to participate in the study was given by the patients.

All patients who were surgically treated for tibial plateau fractures were identified by querying the hospitals databases using the International Classification of Disease (ICD) code. We have formed two separate groups according to our predefined inclusion and exclusion criteria (Table 1).

**Table 1:** Inclusion and exclusion criteria

Inclusion criteria	Exclusion criteria
Acute complex closed intraarticular proximal tibial fracture Type B3, C1, C2, C3 to AO/OTA [19]	Open fractures
	Fractures of type A, B1 and B2 to AO/OTA
Age ≥ 18 years	Polytraumatized patients (Injury Severity Score >16)
Use of intraoperative 3D and 2D imaging intensifier	Foreign residents
Follow-up ≥ 12 months	Conservative treatment
Capable of consent	Pathologic fractures

Study group (3D): Patients who underwent open reduction and internal fixation between December 2015 and December 2018 using 2D and 3D imaging intensifier (Ziehm Vision RFD 3D, Fa. Ziehm Imaging, Nuernberg, Germany).

Control group (2D): Patients who were operated between 2005 and 2014 only by 2D fluoroscopy without 3D imaging.

We retrospectively evaluated the intraoperative revision rate, reasons for revision and operation time of both groups. Additionally, a minimum 12-month postoperative functional outcome was measured by using the KOOS and Lysholm Score [19–21]. Complications, postoperative revision surgeries and infections were analyzed.

Operation procedure: After fracture reduction, including reconstruction of the articular surface and placement of subchondral screws, 2D fluoroscopy was performed in the lateral and anterior- posterior plane. In contrast to the 2D group, an intraoperative 3D scan was now performed in the 3D study group. Whenever possible, as little osteosynthesis material as possible was placed close to the joint surface in order to keep possible artifacts to a minimum for the 3D scan. The decision for re-positioning the screws and/or redo the reduction maneuver was made by the attending surgeon. Particularly intraoperative revision reasons were an intra-articular screw position or a joint step of more than 2mm.

In order to analyze the effect of intraoperative 3D imaging we compared the outcomes of the study group (3D group) to the historical collective (2D group).

We characterized our two groups by means of descriptive statistics: Mean ± standard deviation (SD) for continuous and number (%) for categorical variables.

Both groups were compared by a one to one matching of the patients. The matching criteria were age ± 5 years, exact fracture classification by AO/OTA, gender and American Society of Anesthesiologists- Score (ASA). If no matching partner was found, the ASA score was changed by one unit (n=4). If even then no suitable partner was found, gender (n=1) or gender and ASA (n=2) was adapted. During the matching process, attention was also paid to the follow-up time. The matching patient with the next possible follow-up time to the matching partner was selected. Further analyses were performed by using the Mann-Whitney-U test for non-parametric data (two- tailed test, p- value < 0.05). The software SPSS (V.25, Fa. IBM, New York, USA) was used for the calculations and graphical presentations of the results.

## Results

A total of 22 patients with AO/OTA B3 and C tibial plateau fracture were found according to our inclusion criteria. Four patients were excluded due to different circumstances: no follow-up could be determined for two patients, one patient died before the 1-year follow-up and one patient suffered a polytrauma. Finally, 18 patients were included in our 3D study group, of which 12 were women (Table 2). The mean age was 51.0 ± 16.4 years (range 23–85 years). We had 9x B3, 2x C1, 1x C2 and 6x C3 fractures according to AO/OTA. The mean ASA score was 1.44± 0.51. The mean operation time was recorded with 128± 46 min. The mean follow-up time was 20.9± 10.7 months with the outcome scores shown in Table 3. Postoperative revisions had to be performed in 27.8% of cases. In one case a superficial wound revision was necessary due to a wound healing disorder, in one case we observed a deep surgical site infection with removal of the material and implantation of an unicondylar knee prothesis in the follow-up. One patient required a varus osteotomy due to posttraumatic valgus misalignment and one patient required a renewed reposition because of loss of reduction (Table 4).

**Table 2:** Comparison of 3D study group and matched 2D control group: Age, Gender, Trauma mechanism, ASA- Score, AO/OTA, Operation time, intraoperative Revision

Case	Age	Gender	Trauma mechanism	AO/OTA	ASA	Operation time (min)	Intraoperative Revision	
							YES/ NO	Type of Revision
1	65	f	fall	C3	2	151	YES	Joint step > 2mm, renewed reposition
^m^1	60	f	fall	C3	2	121		
2	60	m	accident at work (machine)	C3	2	214	NO	Anterior fragment <2mm, no consequent
^m^2	63	m	fall	C3	2	167		
3	43	m	motorcycle	C3	1	151	NO	-
^m^3	42	m	accident at work	C3	1	142		
4	60	m	fall down stairs	B3	2	86	NO	-
^m^4	58	m	skiing	B3	2	115		
5	61	f	biking	C1	1	191	NO	-
^m^5	56	m	biking	C1	1	111		
6	52	f	biking	B3	2	94	NO	-
^m^6	47	f	biking	B3	2	71		
7	37	m	biking	C3	1	184	YES	Joint step >2mm, cortical bone chip, reposition
^m^7	34	m	fall down from ladder	C3	1	243		
8	68	f	fall	C2	2	131	YES	Anterior joint step 3-4mm, renewed reposition
^m^8	66	f	fall	C2	2	77		
9	65	f	skiing	B3	2	64	NO	-
^m^9	70	f	fall	B3	2	86		
10	51	f	skiing	B3	1	47	NO	-
^m^10	54	f	fall	B3	2	119		
11	36	f	skiing	C3	1	132	NO	-
^m^11	37	m	biking	C3	2	135		
12	56	f	biking	B3	1	134	YES	Joint step >2mm lateral, renewed reposition,
^m^12	54	f	motorcycle	B3	2	76		
13	23	m	motorcycle	B3	2	155	YES	Negative dorsal slope, joint step >2mm, reposition
^m^13	27	m	fall	B3	1	141		
14	85	f	fall	B3	2	81	NO	Anterolateral joint step < 2mm, no consequence
^m^14	80	f	fall	B3	2	75		
15	38	f	biking	C3	1	95	NO	-
^m^15	43	f	biking	C3	2	104		
16	29	f	biking	B3	1	90	NO	Joint step < 2mm, no consequence
^m^16	33	f	fall	B3	1	77		
17	30	m	biking	C1	1	143	NO	Lateral joint step < 2mm, no consequence
^m^17	26	f	traffic accident	C1	2	170		
18	59	F	skiing	B3	1	159	YES	Joint step > 2mm, renewed reposition
^m^18	57	f	biking	B3	1	63		

*ASA* American Society of Anesthesiologists- Score; ^m^ Matching partner from 2D control group

**Table 3:** Comparison of 3D study group and matched 2D control group: Follow UP time, Outcome parameters by Lysholm and KOOS sub scores, Postoperative revision

Case	Follow-up (months)	Lysholm	Symptoms	Pain	ADL	Sport/Rec	QOL	Postoperative Revision/ time after prim. surgery
1	40	99	96.4	97.2	100.0	85.0	100.0	-
^m^1	42	99	85.7	94.4	97.1	55.0	75.0	-
2	36	48	75.0	66.7	64.7	65.0	43.8	SSI, 1 month: spacer, 6 months: unicondylar KTEP
^m^2	38	69	96.4	88.9	88.2	0.0	75.0	Screw intraarticular without operative consequence
3	34	69	46.4	86.1	85.3	60.0	50.0	-
^m^3	35	58	50.0	58.3	72.1	10.0	18.8	14 months: Arthrolysis and material removal / intraop. screw intraarticular
4	31	87	100.0	80.6	82.4	60.0	31.3	-
^m^4	31	86	100.0	80.6	97.1	90.0	62.5	17 months: Arthrolysis and material removal
5	36	99	92.9	100.0	100.0	85.0	87.5	5 months: superficial wound revision, 19 months: arthrolysis, material removal
^m^5	32	57	64.3	69.4	55.9	0.0	25.0	17 months: Arthrolysis and material removal
6	32	37	57.1	52.8	64.7	25.0	31.3	7 months: arthrolysis, material removal due to pain
^m^6	97	24	14.3	2.8	10.3	0.0	0.0	15 months: Arthrolysis and material removal// intraop. joint step > 2mm
7	25	34	39.3	33.3	41.2	15.0	18.8	-
^m^7	24	46	46.4	66.7	67.7	0.0	25.0	13 months: Arthrolysis and material removal// intraop. joint step > 2mm
8	17	79	100.0	100.0	100.0	55.0	100.0	-
^m^8	133	33	64.3	58.3	60.3	0.0	18.3	Valgus gonarthrosis: after 20 months: Bicondylar KTEP
9	15	90	96.4	97.2	100.0	100.0	93.8	-
^m^9	25	73	96.4	75.0	86,8	0.0	31.3	Postop CT: screw too long without operative consequence
10	15	71	71.4	97.2	98.5	80.0	43.8	-
^m^10	130	60	53.6	83.3	77.9	90.0	6.3	Eight times revision: osteomyelitis/ infect; after 20 months hemiprothesis
11	12	28	28.1	63.9	63.2	5.0	25.0	posttraumatic valgus malalignment, varus osteotomy after 11 months
^m^11	82	74	92.9	86.1	95.6	45.0	12.5	posttraumatic valgus malalignment
12	12	36	50.0	61.1	77.9	0.0	31.3	9 months: valgus deformity, loss of reduction-> reposition
^m^12	39	31	28.6	36.1	42.7	20.0	31.3	17 months: Arthrolysis and material removal (valgus deformity)
13	12	88	67.9	83.3	94.1	95.0	81.3	-
^m^13	42	64	82.1	83.3	92.7	0.0	37.5	-
14	12	95	100.0	100.0	95.6	95.0	100.0	-
^m^14	35	100	100.0	100.0	100.0	40.0	100.0	-
15	12	62	71.4	63.9	66.2	45.0	62.5	-
^m^15	37	69	78.6	86.1	98.5	25.0	37.5	1 month: loss of reposition/ renewed reposition; 20 months: Arthrolysis and material removal
16	12	89	89.3	97.2	97.1	95.0	100.0	-
^m^16	32	55	50.0	75.0	89.7	40.0	40.0	21 months: Arthrolysis and material removal
17	12	17	14.3	30.6	44.1	0	18.8	-
^m^17	77	74	92.9	83.3	91.2	65.0	43.8	18 months: Arthrolysis and material removal
18	12	79	60.7	77.8	85.3	50.0	50.0	-
^m^18	69	44	60.7	63.9	88.2	0.0	50.0	-

*Symptoms* KOOS Symptoms, *Pain* KOOS Pain, *ADL* KOOS Function in daily living, *Sport/Rec* KOOS Function in sport and recreation, *QOL* KOOS Quality of Life; *SSI* Surgical site infection, ^m^ Matching partner from 2D control group; *intraop*. intraoperative

**Table 4:** Parameters of interest for comparison of the 3D and 2D groups

Parameter	3D group	2D group	***p***-Value
Age	51.0± 16.4	50.3± 15.2	0.82
Gender	12f : 6m	11f : 7m	0.79
Fracture AO/OTA	9x B3, 2x C1, 1x C2, 6x C3	9x B3, 2x C1, 1x C2, 6x C3	1.00
ASA	1.44± 0.51	1.67± 0.49	0.27
Operation time (min)	127.9± 45.9	116.3± 45.7	0.28
Follow - up (months)	20.9± 10.7	55.5± 34.7	*P* < 0.001
Lysholm	67.4± 26.8	62.0± 21.4	0.39
KOOS overall	72.6± 23.5	65.8± 21.6	0.31
Symptoms	69.8± 26.6	69.8± 25.8	0.94
Pain	77.2± 22.6	71.8± 23.0	0.44
ADL	81.1± 19.5	78.4± 23.7	0.70
Sport/Rec	54.7± 35.0	26.7± 31.6	0.014*
QOL	58.0± 31.6	38.2± 26.1	0.059
Intraoperative revision rate	33.3% re-positioning of the screws and/or re-reduction	Not registered	-
Postoperative revision rate	27.8% revision (n=5): - wound revision - SSI, spacer, unicondylar TEP - varus osteotomy due to valgus alignment - renewed reposition - early material removal	27.8% revision (*n*=5): - 1x screw intraarticular, material removal - 2x loss of reposition/ renewed reposition - bicondylar KTEP - SSI/ unicondylar KTEP	1.00
Postoperative revisions due to intraarticular material position or joint step of ≥ 2mm	11.1% (*n*=2): - varus osteotomy due to valgus malalignment - 1x loss of reposition/ renewed reposition	22.2% (*n*=4): - 1x screw intraarticular, material removal - 2x loss of reposition/ renewed reposition - 1x loss of reposition/ bicondylar KTEP	0.58
Material removal (months)	1x arthrolysis and material removal after 19 months	10x arthrolysis and material removal after 16.9± 2.6 months	-
Special details without revision	-	2xPostoperative recognized joint step > 2mm 1xpostoperative recognized intraarticular screw 1xpostoperative recognized too long screw	-

*SSI* surgical site infection; *Symptoms* KOOS Symptoms**;** *Pain* KOOS Pain**;** *ADL* KOOS Function in daily living**;** *Sport/Rec* KOOS Function in sport and recreation**;** *QOL* KOOS Quality of Life**;** *ASA* American Society of Anesthesiologists- Score

* significant

Due to the 3D imaging an intraoperative new reduction or new placement of the implants had to be performed in 33.3% (6/18).

In one case, the intraoperative 3D imaging showed insufficient quality to adequately control the fracture reduction and implant positioning. All other 17 cases (94.4%) showed good quality according to the surgical reports.

According to the matching criteria, 18 patients of the 2D control group could be matched in a ratio one to one (2D group: mean age: 50.3± 15.2years; 11 females). Due to the matching both groups are comparable in terms of age, gender and ASA score (p> 0.05). The mean follow-up time of the 2D group was 55.5± 34.7 months (p< 0.001). The functional outcome scores of the 3D study and 2D control group are shown in Table 3. Especially the KOOS Sport/Rec sub score showed significantly higher results for the 3D group. The rate of postoperative revisions of the 2D group was 27.8 %. An infection has been observed in one case. The duration of the operations was 116± 46 min (p=0.28).

If postoperative CT control imaging was performed in the 2D control group due to pain, one case showed intra-articular screw position and two cases showed joint steps > 2mm without any operative revision. In the 3D study group no postoperative intraarticular implants or joint steps >2mm were detected.

## Discussion

The hypothesis of the study that the additional use of intraoperative 3D imaging results in a relevant number of intraoperative revisions (33.0%), potentially leading to a significantly improved outcome (KOOS Sport/Rec) could be approved. In the first analysis, this did not lead to a reduction in postoperative revisions. This might be due to the fact that in the 2D control group, relevant joint steps and suboptimal material position were tolerated in the presence of mild clinical symptoms (22.2%).

The patients in both groups were about 50 years old and are comparable based on the matching process except for the duration of the follow-up time. Based on previous studies, the duration of follow-up should be evaluated subordinately, as previous studies could show that the outcome of the TPF is stable in the medium-term period of up to ten years after trauma [22, 23].

The outcome between 3D group and 2D group are comparable with respect to Lysholm, KOOS Symptoms, KOOS Pain and KOOS ADL. The sub scores of KOOS Sport/Rec showed significantly better results in 3D group and a trend to higher KOOS Quality of Life scores (p=0.059). These subscales reflect complaints of the knee joint under higher loads, stress and more demanding movements. It is therefore conceivable that the optimized joint reduction after 3D imaging is not of high relevance for everyday activities and pain, but is particularly evident under physical stress. In the KOOS subscales for everyday movements, symptoms, and pain no significant differences could be shown between the both groups.

The majority of fractures in this study were severe joint injuries, B3 and C3 fractures according to AO/OTA. This explains lower scores compared to other studies [22, 24]. However, Jansen et al. who included patients with similar injury severity (C1-3 fractures according to AO/OTA) reported comparable results to our study (Lysholm 66.2; KOOS overall 67.8) [25]. Additionally, better results can be expected in patients with type B1, B2, and C1 fractures compared to B3 and C3 fractures [22, 26, 27].

A major advantage of this study should be not only to record the outcome of patients operated with 3D imaging in a minimum follow-up of 12 months, but also the comparison with patients operated without this technique. We decided to use matching as a method of comparison, as only the image intensifier was modified throughout the course of time. For this, we had to accept the limitation of the longer follow-up of the control group, with the advantage of no further influencing factors.

Main matching criterion was the same fracture morphology (B1, B2, B3, C1, C2, C3) according to AO/OTA, followed by the age ± 5 years. These criteria were always met. In order to show the bone metabolism and the patient's state of health, the importance of gender and the ASA score followed.

The rate of intraoperative revisions due to the used 3D scan is with 33.3% higher than in other studies (17.2± 6.1%; range 11.7- 26.5% [14–18, 27]). The reasons for this are different revision criteria.

Based on the discussed parameters for joint gap with values between 1-4 mm, we decided on a revision, if the joint gap is more than 2 mm [6, 8, 9, 28–31]. An intraarticular or overlong screw certainly causes symptoms. The most common fractures requiring intraoperative revision were B3 and C3 fractures (71.4 %), which can be explained by the severely destroyed, multifragmentary articular surface and the split depression component.

For this 2D fluoroscopy has its limitations due to the concave anatomical joint conditions of the proximal tibia, with the result that joint steps < 5 mm cannot be adequately detected with fluoroscopy [11, 32, 33]. Conventional fluoroscopy is highly dependent on the set plane, while 3D imaging provides image information regardless of the position of the device [34, 35].

Due to the high rate of intraoperative revisions, our secondary hypothesis was that the rate of relevant postoperative revisions can be reduced by using 3D scan. Unfortunately, this cannot be confirmed. Both the study group and the control group show a postoperative revision rate of 27.8%.

In further analysis of the 2D group, postoperative CT was performed in some patients. In two cases joint steps of more than 2mm, in one case an intraarticular screw and in one case a too long screw were detected but accepted without revision (22,2%). The estimated number of unreported cases may be higher. The detected 4 patients with revision criteria are the reason why there are no higher revision rates in the 2D group, but could probably be the reason for the poorer functional results of the 2D group in KOOS Sport/Rec and QOL.

There were no significant group differences between the 3D and 2D groups except the mentioned longer follow-up time of the control group. Nevertheless, a prolonged surgery time of approximately 12 minutes on average was observed for the study group, which was due to the use of intraoperative 3D imaging and performed intraoperative revisions in 33.3% of cases. Beisemann et al. also describe longer operation time by using intraoperative 3D scan (5 min) [16]. In recent years, numerous studies have been published on the use of intraoperative 3D imaging of joint injuries (proximal humerus, distal radius, proximal and distal tibia fractures). In most cases, acceptable image quality was shown for the assessment of an insufficient joint level or a material defect with high revision rates [15, 16, 36–39]. Overall, the technology of intraoperative 3D imaging remains reserved for the use of large clinics due to high acquisition costs.

### Limitations

Due to the group comparison by matching according to the specific criteria, small differences in the ASA score (five cases), one for gender and one for both criteria together had to be tolerated. In this way a matching in the ratio one to one was possible between the groups. By softening the matching parameters, a matching ratio of 1:2 between the intervention group and the control group could certainly have been achieved. However, this was not desired. Also, the follow-up time varied between the 3D group and the 2D group. However, based on existing publications of the medium and longer follow-up time, the time of determination of the functional outcome should not have much influence, since it is below 5 years in both groups [22, 23]. Due to the retrospective study design, we had to rely on the data available in medical records (e.g. operation protocols). Unfortunately, not all intraoperative 3D scans were stored in the database, so that a later evaluation of the image quality of the 3D scan was not possible. It was necessary to rely on the documentation. Additionally, the 3D study group was compared with a historical control group. Nevertheless, the factors to be monitored, such as the implants, the surgical approach, general anesthesia and the postoperative physiotherapeutic regime (with 20 kg of partial weight-bearing for 6 weeks under free range of motion), did not change during this period. Tranexamic acid was not used in any of the groups. However, a certain selection bias that developed over time cannot be excluded with certainty. Due to the retrospective character, the decision for revision was made by the responsible surgeon. A possible learning curve in the supply of a 3D scan must remain suspected, which might hide additional advantages of the 3D scan. A further deficiency is that the patients of the study group and the control group were treated by different surgeons.

## Conclusion

The intraoperative 3D imaging had in relevant number an immediately intraoperative consequence with revision. An influence of intraoperative 3D imaging on the midterm postoperative outcome could be shown in relation to better KOOS Sport/Rec.

## Data Availability

The datasets used and/or analyzed during the current study are available from the corresponding author on reasonable request.

## Abbreviations

ASA: American Society of Anesthesiologists
AO/OTA: Arbeitsgemeinschaft Osteosynthese/Orthopaedic Trauma Association
CT: Computer tomography
KOOS: Knee Injury and Osteoarthritis Outcome Score
TPF: Tibial plateau fractures
